# Safety and Efficacy of Long-Acting Insulins Degludec and Glargine Among Asian Patients With Type 2 Diabetes Mellitus: A Meta-Analysis

**DOI:** 10.7759/cureus.16046

**Published:** 2021-06-29

**Authors:** Ravi Kant, Poonam Yadav, Mohit Garg, Yogesh Bahurupi, Barun Kumar

**Affiliations:** 1 General Medicine, All India Institute of Medical Sciences, Rishikesh, IND; 2 College of Nursing, All India Institute of Medical Sciences, Rishikesh, IND; 3 General Medicine, Government Medical College, Khandawa, IND; 4 Community and Family Medicine, All India Institute of Medical Sciences, Rishikesh, IND; 5 Cardiology, All India Institute of Medical Sciences, Rishikesh, IND

**Keywords:** glycemic control, hypoglycemia, insulin degludec, insulin glargine, long-acting insulin, type 2 diabetes

## Abstract

Background

Global variation in susceptibility to diabetes, insulin sensitivity, and regimen intensity poses a challenge for clinicians regarding the optimal choice of insulin therapy. The current study was carried out to see the relative safety and efficacy of currently available long-acting insulins among the type 2 diabetic Asian population.

Methods

A systematic literature search was done using various search engines (PubMed, Cochrane, Google Scholar, Scopus, and Embase) and included published randomized controlled trials (RCTs) in English before December 2019. Further, a manual search was performed by screening the reference list of the identified articles.

Results

We included four RCTs with 534 participants (349 in the insulin degludec group and 185 in the insulin glargine group) with type 2 diabetes mellitus (T2DM). Results show that both insulin glargine and degludec are equally efficacious in reducing fasting blood glucose (mean difference is -4.45, confidence interval -13.32- 4.43, I^2^=67%) and HbA1c (glycosylated hemoglobin) (mean difference is 0.12, confidence interval -0.12-0.35, I^2^=0%). However, insulin glargine was associated with lower risks of hypoglycemia (risk ratio = 0.9684, confidence interval- 0.8003- 1.1717, I^2^=30%).

Conclusion

Insulin glargine and degludec are comparable in achieving glycemic control with fewer hypoglycemic episodes in the insulin glargine-treated group.

## Introduction

Type 2 diabetes mellitus (T2DM) is a chronic non-communicable disease characterized by progressive B- cell dysfunction [1]. It occurs due to hyperglycemia and insulin resistance leading to increased insulin demands of tissue that culminates into over-functioning of B-cells and ultimately leading to B-cell failure [2].

According to International Diabetes Federation's *IDF Diabetes Atlas 2019*, globally, 463 million people live with diabetes mellitus, and out of that, 88 million are from the South East Asia region (SEAR) only. By 2045, it is expected to increase to 51% globally and 74% in SEAR [3]. Race and ethnic differences variate the susceptibility to diabetes, insulin sensitivity, and regimen intensity which poses a challenge regarding the optimal choice of second-line therapy for clinicians [4-6]. The Asian population is at higher risk of developing type 2 diabetes mellitus (DM) than the European population and develops diabetes at a lower BMI [7-9]. In the South-East Asian diabetic population, 90% of the population has T2DM, which is preventable [10].

Glycemic control is the only way to reduce microvascular and macrovascular complications of DM, which can be attained by effective pharmacotherapeutic agents [11]. Currently, available treatment options include oral antidiabetic drugs and insulin. Oral antidiabetic drugs are often limited in their efficacy to reduce HbA1c beyond 1-2% [12]. Insulin is the only drug that can reduce HbA1c to exceptionally lower levels and maintain it near normal. [13]. Newer long-acting insulins are more stable and efficacious than conventional insulin [14].

There are conflicting results in earlier studies like a meta-analysis by Su W et al., which found that glargine was superior to degludec [15]. Contrary to this, a meta-analysis by Zhou W et al. found a degludec much better than glargine [16]. In view of the conflicting data regarding safety and efficacy, it is required to compare and prioritize the treatment so far as long-acting insulin use is concerned. It was altogether more important to undertake studies in the Asian population as earlier meta-analyses hardly included this population. This study aimed to evaluate the safety and efficacy of currently available long-acting insulin (insulin degludec and glargine) in T2DM Asian patients.

## Materials and methods

Data sources with search strategy

**Figure 1:**
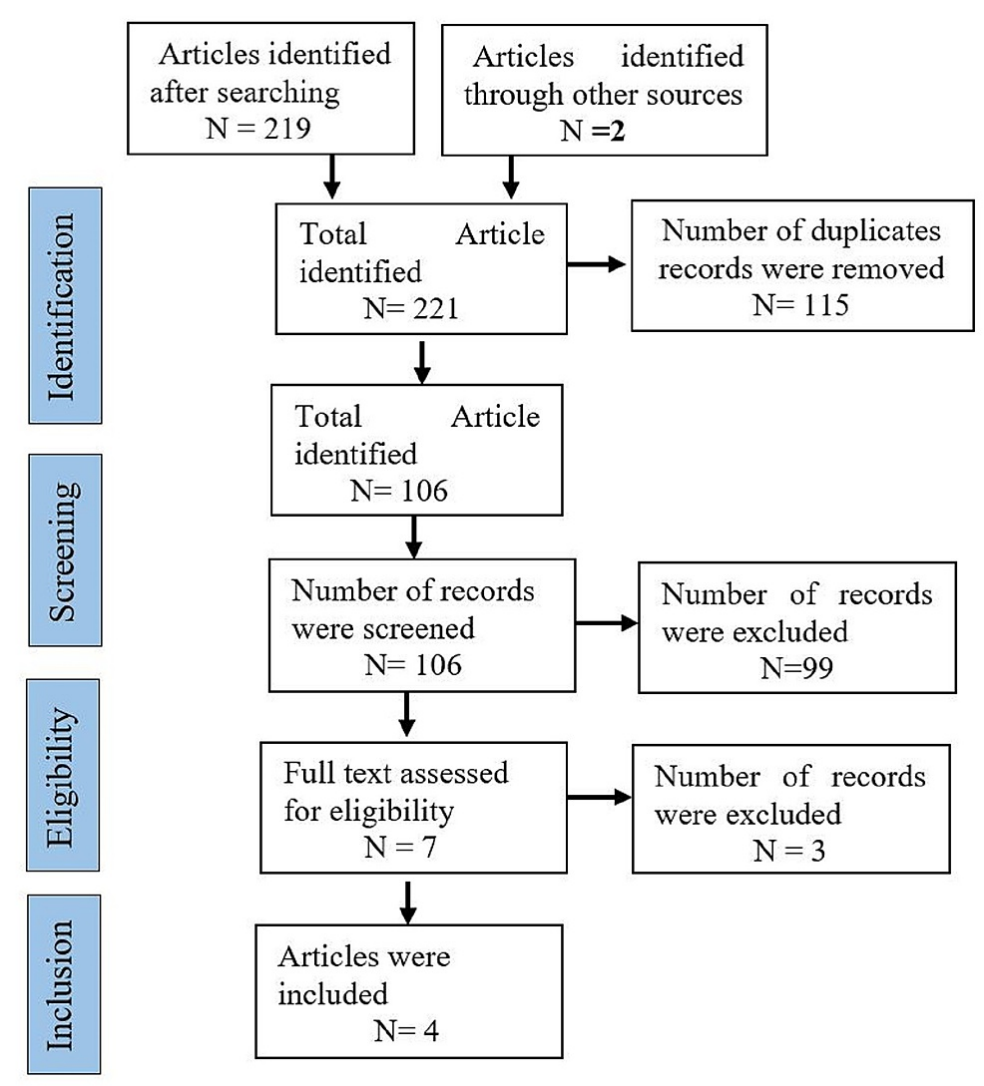
PRISMA flow chart PRISMA: preferred reporting items for systematic reviews and meta-analysis

A systematic literature search was done as per the PRISMA protocol (preferred reporting items for systematic reviews and meta-analysis) [17] (Figure 1) and PICO (participant, intervention, comparison, and outcome) format.

Our search strategy included all relevant published randomized controlled trials (RCTs) before December 2019 from PubMed, Cochrane, Google Scholar, and Embase. Articles in the English language and having an Asian population were included. Keywords that were used for the search were (“insulin Degludec” OR “degludec” OR “IDeg”) AND (“insulin glargine” OR “insulin” “glargine” OR “IGlar”) AND (“randomized” OR “randomly”) AND (“diabetes” OR “diabetes mellitus” OR "type 2 diabetes mellitus, OR “type 2 diabetes”) AND (“Asians”). Further, a manual search was performed by screening the reference list of the identified articles.

Inclusion criteria

1) Patient population: All studies enrolling Asian type 2 diabetes patients

2) Intervention: insulin degludec

3) Comparison: insulin glargine

4) Study design: RCTs (parallel-group and cross-over study designs)

5) Outcomes: Safety of insulins was assessed with a risk of hypoglycemia (episodes >1) of documented hypoglycemia (<70 mg/dl) and efficacy was assessed by improvement or change in the HbA1c or fasting plasma glucose

Exclusion criteria

1) Articles on animal studies and studies conducted outside Asia

2) Review articles, observational, quasi-experimental studies, and case series

3) Studies with less than 12 weeks duration of intervention

Data extraction

Two reviewers (PY and MG) screened the studies for eligibility and data extraction independently. Any disagreements were resolved by discussion with a third reviewer (RK). The data were extracted from eligible studies without modification of original data onto pre-existing data collection forms: study location, year of publication, sample size, types of sample selection and allocation to two groups (intervention and comparator or control group), methods, types of administration of intervention and control, and primary outcomes. Authors of selected articles were contacted for the required data for analysis. The search file was imported into Zotero library (Center for History and New Media, George Mason University, Fairfax County, USA). After removing duplicate items, to exclude studies, we used Rayyan (Qatar Computer Research Institute, Doha, Qatar) [18].

Evaluation of the risk of bias (quality) assessment

The risk of bias has been evaluated with the Cochrane Risk of Bias Assessment Tool. Two reviewers (PY and MG) assessed the risk of bias independently, and discrepancy at any point was also discussed with the third reviewer (YB) also. The risk of bias assessment has been calculated undertaking blinding (masking) of participants, researcher and outcomes, allocation concealment, randomization details, data outcome, and data reporting [19]. Quality assessments have been done with GRADEpro GDT software (McMaster University and Evidence Prime, Inc., Hamilton, Canada). Relevant file has been imported from RevMan software (Cochrane Training, London, UK) to the "Summary of Findings" table GRADE Profiler to create a "Summary of Findings" table [20]. The summary of the intervention effect and a measure of the quality of evidence was noted in the table.

Data analysis

Four RCTs that suit the eligibility criteria were enrolled [21-24]. Onishi et al. considered the confirmed hypoglycemia as plasma glucose level <3.1 mmol/L or 55.86 mg/dl and found lower hypoglycemia rates in degludec treated patients as opposed to the glargine group [23]. These findings are reinforced by Aso Y et al., with a similar improvement in glycemic control in both groups but a low risk of hypoglycemia with degludec, considering plasma glucose level as <70 mg/dl for confirmed hypoglycemia [21]. In contrast, Kawaguchi Y et al. [22] and Yamabe M et al. [24] considered the confirmed hypoglycemia as plasma glucose level <70 mg/dl and observed that insulin glargine and insulin degludec are equivalent long-acting insulin analogs and insulin glargine had lesser episodes of hypoglycemia. They noted the mean percentage of time in the target glucose range (70-179 mg/dL) and considered hypoglycemia as <70 mg/dL, with flash glucose monitoring weekly. Authors of two studies, Kawaguchi Y et al, 2019 [22] and Yamabe M et al. 2019 [24], have been contacted for the number of events with hypoglycemia episodes and post-intervention data for change in HbA1c (mean and standard deviation) for the relevant data required in the meta-analysis, but the unavailability of data reinforces the decision to exclude these studies from the final analysis.

Relevant findings were tabulated and displayed based on the forest plots. The I^2^ statistic was used to test statistical heterogeneity, which may arise due to inter-trial variability, with >75% values representing important heterogeneity with a fixed-effects model (95% confidence interval, p-value < 0.05). For calculating efficacy, the mean difference between the insulin degludec and glargine group was calculated to show differential mean changes in HbA1c and fasting plasma glucose (FPG) levels. For calculating safety between insulin degludec and glargine group, the risk ratio (RR) was calculated. We have not observed significant heterogeneity for hypoglycemia and glycemic control incidence. Sensitivity analysis was performed to control fasting plasma glucose, although we observed similar heterogeneity with a random-effect model. Subgroup analysis could not be possible due to the limited number of studies.

## Results

The Meta-analysis was performed using the RevMan version 5.4 software. Finally, four RCTs with a total of 534 participants (349 in the insulin degludec group and 185 in the insulin glargine group) with T2DM were eligible for inclusion in this meta-analysis. Characteristics of participants in each trial are described in Table 1.

**Table 1:** Characteristics of participants in each trial

Author and year of the study	Setting	Population	Number of Participants	Duration of Trial	Mean Age of Participants	Duration of Diabetes	Gender Distribution
Aso Y et al., 2017 [21]	Single-Center Study	Japan	45 participants [33 in insulin degludec group and 12 in glargine group]	24 weeks	64.0±13.6 in insulin degludec and 64.7±15.7 in insulin glargine	10 years in degludec and 13 years in insulin glargine	Female participants 55%
Kawaguchi Yet al., 2019 [22]	Single-Center Study	Japan	30 patients [15 patients in each group]	6 months	69.5 ± 11.3 years	18.3 ± 11.3 years	Male participants (60%)
Onishi Y et al., 2013 [23]	A Multi-center Study	Hong Kong, Japan, Malaysia, South Korea, Taiwan, and Thailand	435 participants [289 in insulin degludec group and 146 in glargine group]	26 weeks	58.6 years	11.6 Years	Male participants (53.56%)
Yamabe M et al., 2019 [24]	Single-Center Study	Japan	24 participants [12 in each group]	5 months	70.7 ± 7.6 (years)	14.0 ± 9.3 (years)	Male (50%)

The summary of findings with the grade of evidence are shown in Table 2. This table has displayed the certainty of evidence and grade as high, moderate, and low for each outcome variable.

**Table 2:** GRADEpro summary of findings ^a^heterogeneity; ^b^limited studies; ^c^wide confidence interval; *significant effect; ⨁◯ symbols indicating certainty of evidence CI: confidence interval; MD: mean difference; HbA1C: glycosylated hemoglobin; RR: risk ratio

Patient or population: Type 2 diabetic patients					
Setting: Asian population					
Intervention: Insulin degludec					
Comparison: insulin glargine					
Outcomes	Anticipated absolute effects^*^ (95% CI)		Relative effect (95% CI)	№ of participants (studies)	The certainty of the evidence (GRADE)
	The risk with insulin glargine	The risk with Insulin degludec			
Fasting blood sugar	The mean fasting blood sugar was 0	MD 4.45 lower (13.32 lower to 4.43 higher)	-	508 (3 studies)	⨁⨁⨁◯ MODERATE ^a, b^
Decrease in HbA1c	The mean decrease in HbA1c was 0	MD 0.12 higher (0.12 lower to 0.35 higher)	-	480 (2 studies	⨁⨁⨁◯ MODERATE ^b^
Risk of hypoglycemia	506 per 1,000	491 per 1,000 (405 to 592)	RR 0.97 (0.80 to 1.17)	473 (2 studies)	⨁⨁◯◯ LOW ^a, b, c^

**Figure 2:**
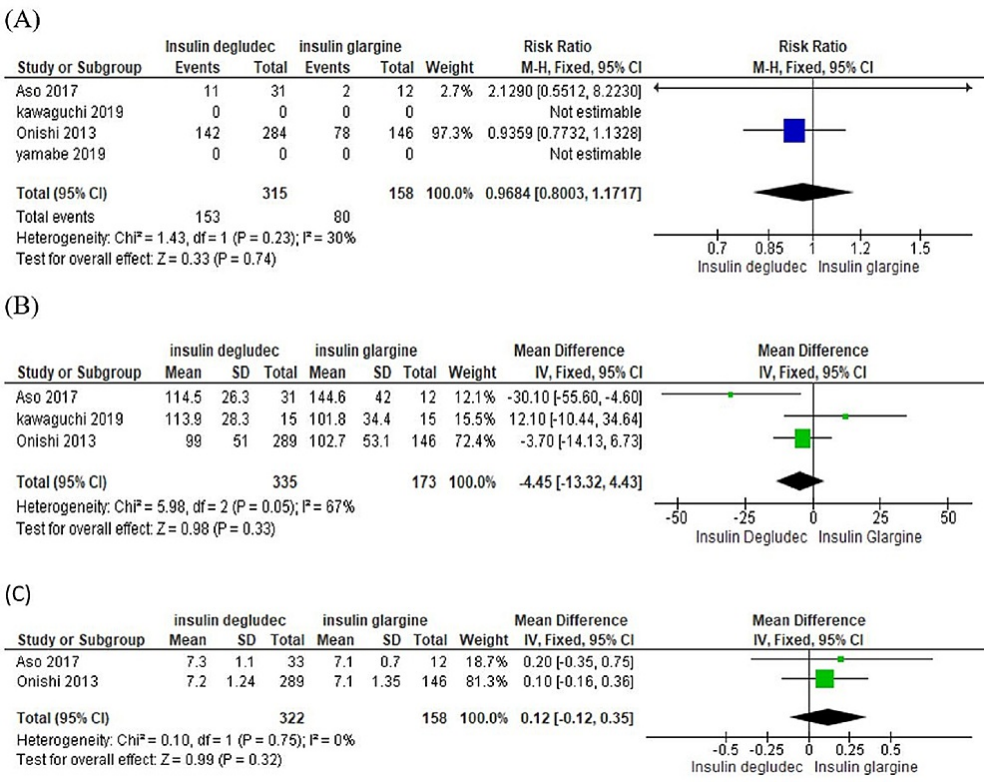
Forest plot shows the comparison of insulin degludec and glargine A)  Incidence of hypoglycemia; B) Control of fasting plasma glucose; C) Decrease in HbA1c CI: Confidence Interval; SD: Standard Deviation [19]

Figure 2A shows the forest plot for hypoglycemia risk with RR = 0.9684, suggesting that hypoglycemia is less with glargine, CI of RR (0.8003- 1.1717) with I^2^=30%, which shows mild heterogeneity among studies.

Figure 2B shows the forest plot which states the no statistically significant difference in fasting plasma glucose between insulin degludec and glargine [mean difference is -4.45, confidence interval -13.32- 4.43] with I^2^=67%, p-value=0.02, which shows heterogeneity among studies.

Figure 2C shows the forest plot which states no statistically significant difference in HbA1c between insulin degludec and insulin glargine [mean difference is 0.12, confidence interval -0.12-0.35], I^2^=0%.

**Figure 3:**
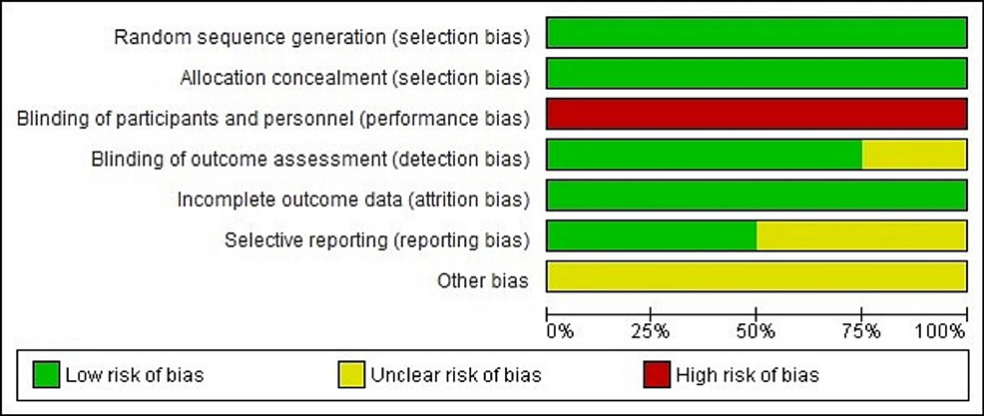
Risk of bias graph Review authors' judgements about each risk of bias item presented as percentages across all included studies [19]

**Figure 4:**
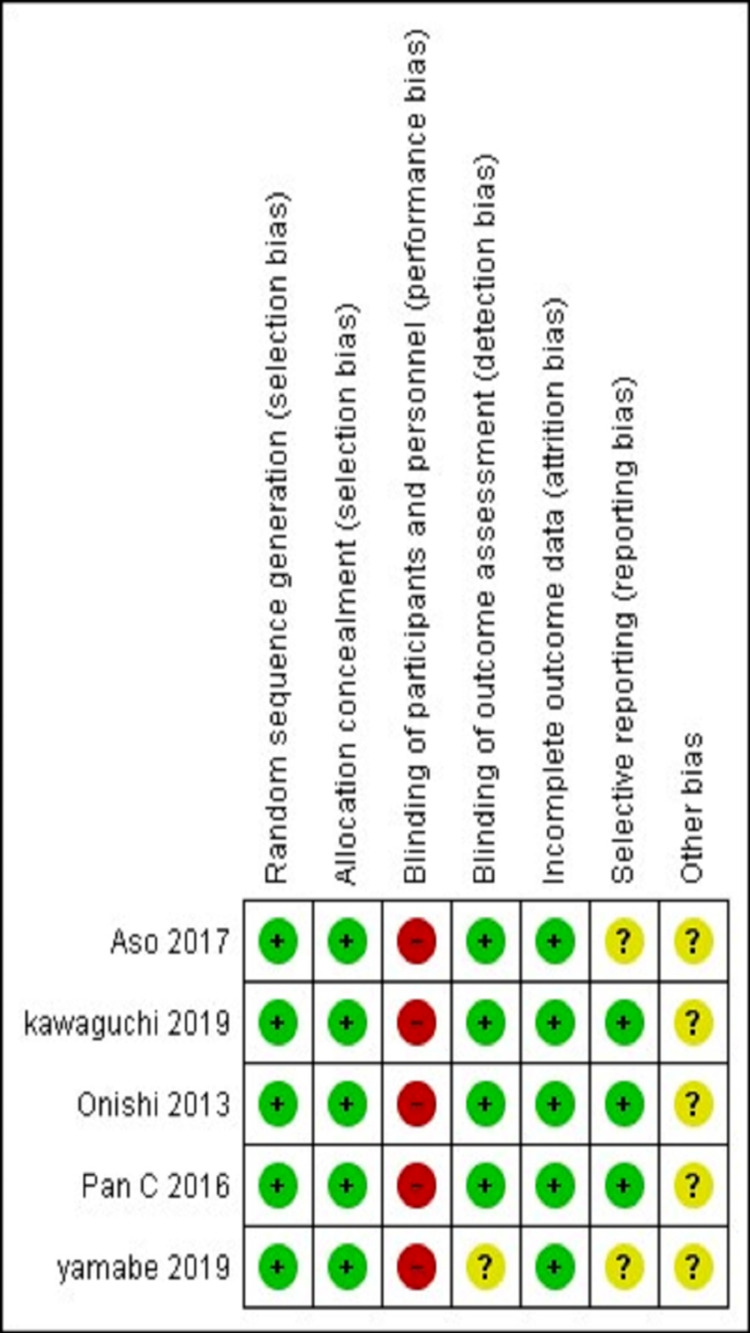
Risk of bias summary Review authors' judgments about each risk of bias item for each included study [19]

The risk of bias graph and summary have been displayed in Figure 3 and Figure 4, respectively, which included random sequence generation, allocation concealment, detection bias, attrition bias, reporting bias, and others in each study.

Funnel plots have not been created due to the limited number of studies in the analysis.

## Discussion

Optimum glycemic control in T2DM patients represents a challenge even with advanced pharmacologic treatment [11]. Evidence suggested that insulin therapy has been convincingly superior in attaining target glycemic control over oral anti-diabetic drugs [12]. However, its use is accompanied by hypoglycemia episodes; long-acting insulin therapy is a safe and effective way of treating diabetes mellitus [13].

In the present meta-analysis, the efficacy and safety of currently available long-acting insulin (insulin degludec and glargine) were compared in type 2 diabetes mellitus Asian patients. A total of four high-quality RCTs were included to evaluate outcome measures. In the present study, both insulins effectively reduced fasting plasma glucose and HbA1c.

We have noted no statistically significant mean difference in fasting plasma glucose (mean difference is -4.45, confidence interval -13.32- 4.43) and HbA1c reduction (mean difference is 0.12, confidence interval -0.12-0.35) between both groups. For comparing safety between the groups, RR = 0.9684, suggestive that risk of hypoglycemia is less with glargine, CI of RR (0.8003- 1.1717) with I^2^=30%. A meta-analysis by Heller S et al. showed a lower rate of hypoglycemia in the degludec group [25]. Findings of this study are supported by Onishi et al. and Pan C et al. both these investigators considered the confirmed hypoglycemia as plasma glucose level <3.1 mmol/L or 55.86 mg/dl and did find lower events of hypoglycemia in degludec treated patients as opposed to glargine group [23, 26]. These findings are reinforced by Aso Y et al. also with a similar improvement in glycemic control with both insulins but a lower risk of hypoglycemia with degludec, considering higher plasma glucose level (<70 mg/dl) for confirmed hypoglycemia [21]. Kawaguchi Y et al. [22] and Yamabe M et al. [24] also considered the confirmed hypoglycemia as plasma glucose level <70 mg/dl and observed that insulin glargine and insulin degludec are equivalent long-acting insulin analogs and insulin glargine has lesser episodes of hypoglycemia. A meta-analysis by Ratner RE et al. also concluded that similar improvements in glycemic control could be achieved along with fewer hypoglycemic episodes with insulin glargine [27]. The findings of Ratner RE et al. and Rodbard HW et al. were conflicting, as later concluded that insulin degludec had fewer hypoglycemic events than insulin glargine [28], although clinical efficacy was similar. Even Russell-Jones D et al. reported a higher clinical safety and efficacy of insulin degludec than insulin glargine among patients with T2DM [29]. A randomized controlled trial among T2DM patients also concluded that insulin degludec improved glycemic control similarly to insulin glargine with a lower risk of hypoglycemia [30].

It is apparent from the studies that most researchers found a lower hypoglycaemic profile in patients treated with insulin degludec. These results could be partially explained by the cut-off value of plasma glucose to define hypoglycemia as a value of 55.86 mg/dl or less than the plasma sugar of 70 mg/dl is expected to exclude a fair number of patients with hypoglycemia. Our findings are contrary to most available literature, as, in the current meta-analysis, we found that patients with insulin glargine were associated with lower rates of hypoglycemia. However, both insulins had equal propensity to achieve short-term and long-term glycemic goals.

This meta-analysis is novel work as there were only a few studies conducted in the Asian population to compare the safety and efficacy of currently available long-acting insulin (insulin degludec and glargine) in type 2 diabetes mellitus patients.

Limitations

Studies included in this meta-analysis were open-label design, and results represent heterogeneity.

## Conclusions

This meta-analysis concluded similar efficacy of currently available long-acting insulin (insulin degludec and glargine) in T2DM patients in the Asian region, with lower hypoglycemia episodes with insulin glargine. It also found a limited number of trials in the Asian population, suggesting the need for future research for comparing the relative safety and efficacy of currently available insulins.
